# Supplementary material for: Are carbon emissions trading and green financial instruments synergistic? -Comprehensive quantitative research based on content analysis
Source: PLoS One. 2024 Mar 7;19(3):e0298601. doi: 10.1371/journal.pone.0298601 (PMC10919601; doi:10.1371/journal.pone.0298601)
Supplement: S1 Data — (ZIP) [file pone.0298601.s001.zip › Data/地级市Z_F工作报告.zip!/╕ú╜¿/┴·╤╥╩╨/└┤╫╘╣1⁄2╓┌║┼í╛┬φ┐╦╩2╛▌═°í┐_2017.docx]

各位代表：

现在，我代表龙岩市人民政府，向大会报告政府工作，请予审议，并请市政协委员提出意见。

一、2016年和过去五年工作回顾

2016年，在省委、省政府和市委的坚强领导下，我市各级政府积极践行新发展理念，扎实推进供给侧结构性改革，全力打好项目落地、脱贫攻坚、生态环保“三大战役”，经济社会保持稳中有进的发展态势。预计全市地区生产总值1891亿元，增长8%；一般公共预算总收入266.1亿元，地方一般公共预算收入131.4亿元、增长5.5%；全社会固定资产投资2245亿元，增长16.2%；社会消费品零售总额729亿元，增长14%；外贸出口160亿元，与上年持平；实际利用外资2.76亿美元，增长6%；内联引资实际利用市外资金210亿元，增长7%；城镇居民人均可支配收入30616元，增长8.5%；农民人均可支配收入14535元，增长9.5%；居民消费价格总水平上涨1.6%；城镇登记失业率2.52%；年度节能减排降碳任务预计可以完成。

主要工作和成效是：

（一）打好“三大战役”，重点攻坚成效明显

项目落地全面提速。滚动实施“五个一批”项目，实行挂图作战、按单销号，推动项目加快落地建设，投资增速保持全省前列。全年新开工建设10亿元以上项目44个、新竣工投产10亿元以上项目37个。省市重点项目共完成投资807亿元，占年度计划的133%。对接六批国家专项建设基金项目138个、争取资金67.6亿元。开展“政民外”重大项目对接活动，落地项目172个、完成投资91亿元。

脱贫攻坚精准扎实。制定脱贫攻坚系列政策，完成贫困户建档立卡，组织市县乡干部挂钩帮扶，逐户落实脱贫措施。组建扶贫开发投融资平台和扶贫小额信贷担保基金，完成造福工程扶贫搬迁3.17万人，帮扶1.92万贫困户各发展1个以上增收项目，实施辅助就业1.75万人。加强兜底保障，提高贫困人口低保标准和新农合报销比例、大病保险报销封顶线。年度脱贫5.5万人，其中国定对象3.45万人。

生态环保有力有效。制定流域环境保护、重点乡镇水质考核管理等措施，全年共投入5.7亿元开展养殖业污染治理，关闭拆除养猪场1.2万户、削减生猪110万头，中心城市内河水质达标率为100%。完成水土流失治理54万亩、造林绿化13万亩，治理废弃矿山82万平方米。淘汰黄标车1万多辆，建成大气污染治理项目12个，城市空气质量优良天数比例达98.6%。

（二）深化供给侧结构性改革，产业转型步伐加快

五大任务全面落实。出台推进供给侧结构性改革措施，“三去一降一补”取得实质性进展。坚定不移去产能，淘汰煤炭产能136万吨、水泥产能30万吨。加快房地产去库存，全年销售商品房275万平方米、增长23%，商品住房去库存期为12个月、比上年下降5个月，处于合理区间。强化债务“控新化旧”，开展非法集资专项整治，全市不良贷款率为1.88%，信贷资产质量居全省较好水平。大力帮扶企业，开展“进企业、送服务、解难题、促增长”活动，为250家企业提供38亿元应急周转资金，为1195家企业提供75亿元贷款担保，减轻企业税费负担26.5亿元。策划实施两批29个投资工程包，完成投资283亿元，加快补齐基础设施、产业发展、社会事业、民生保障等领域短板。

产业结构进一步优化。实施产业兴市战略，推进"365"产业集聚发展。九大工业重点产业实现产值1968亿元、增长11.2%，光电与新材料、生物医药等新兴产业产值增长20%以上，高新技术产业增加值增长10.5%。特色农业加快发展，闽西“八大珍”“八大鲜”产值增长34%。文化旅游、商贸物流等现代服务业提速发展，探索形成农村电商“龙岩模式”。全市第三产业增加值增长10.1%，所占比重比上年提高1.3个百分点。

新动能加快孕育。互联网+、矿业+等新模式、新业态加快兴起，龙马环卫“制造业+服务”模式逐步在全省、全国推广，龙净环保从产品制造向工程总包拓展。大力推进创业创新，新认定省级众创空间5家，市科技创业园获批为国家级科技企业孵化器。军民多领域合作模式正加快形成，全年与部队、军工企业新签约项目52个、总投资330亿元，民品参军销售额超过10亿元，成功创建省级军民融合新型工业化产业示范基地。

（三）推进新型城镇化，城乡环境持续改善

基础设施日益完善。研究出台综合交通一体化规划。浦梅铁路建宁至冠豸山段、厦蓉高速龙岩东联络线、古武线永定至上杭高速公路、小池至古田快速通道等项目开工建设，中心城区至高新园区快速通道、双永高速龙岩北互通连接线等项目建成通车。完成国省干线建设改造90公里，农村公路提级改造、生态路建设230公里。西气东输正式接通龙岩。

城市功能持续提升。实施中心城市道路畅通、市政提升、旧城改造等重点工程，西外环、龙岩大道南段一期、龙岩大道高架桥等主干道路实现通车或部分通车；黉门前、莲花山交通节点可在2017年春节前实现部分通车；龙岩火车站北站房开工建设，龙津湖片区改造、东肖新城开发等项目有序推进。新增公共停车位2804个，新建人行天桥7座。开通中心城市至永定城区公交线路，积极推动基础设施、公共服务等同城配置。

乡村建设力度加大。实施新型城镇化和美丽乡村建设三年行动计划，124个美丽乡村和133个省级“千村整治、百村示范”工程全部开工建设，农村家园清洁行动深入开展。古田镇列入国家首批特色小镇试点，14个村列入第四批中国传统村落名录，67个村列入全国乡村旅游扶贫重点村。

（四）办好惠民利民实事，社会保持和谐稳定

社会保障更加有力。全市公共财政用于各类民生支出217亿元，增长6.5%，占地方公共预算支出的79%。完成25件为民办实事项目。新增城镇就业2万人，农村劳动力转移就业4.5万人。保障性安居工程新开工1.7万套，基本建成7711套。党政军民合力抗灾救灾，最大限度降低损失，受灾重建户可在2017年春节前全部迁入新居。

社会事业加快发展。农村薄弱学校改造、城区中小学扩容扎实推进，新建公办幼儿园10所。医药卫生体制改革深入推进，公立医院全面实行药品、耗材零差率，“互联网+分级诊疗”正式运营。在全省率先推行医保卡购买健身服务模式。成功举办全国女子举重锦标赛和首届环土楼国际山地马拉松赛。举办纪念红军长征胜利80周年系列活动，反映闽西子弟兵浴血湘江的电视剧《绝命后卫师》在央视热播，十大群众文化活动、文化惠民工程深入开展。

社会治理不断改进。创建全国文明城市成效初显，城市管理、社区建设和市民素质进一步提升。法治龙岩、平安龙岩建设深入推进，涉麻制毒、电信诈骗专项治理得到公安部充分肯定。抓好安全生产重点整治，安全生产形势持续稳定好转。深入开展“餐桌污染”治理，启动建设食品药品安全放心市三年行动，食品药品安全得到有效保障。

（五）加强政府自身建设，行政效能进一步提高

深入开展“两学一做”学习教育，牢固树立“四个意识”，加强党风廉洁建设，严格落实中央八项规定精神，政府作风进一步转变。推进简政放权，实现市县乡三级政府权责清单全覆盖，在全省率先推行投资项目告知承诺预审批，建成全省首个市县乡一体化网上办事大厅。党政机关公务用车改革全面完成。自觉接受市人大及其常委会的法律监督、工作监督和市政协的民主监督，办理人大代表建议279件，办复率、满意率均为100%，办理政协提案573件次，办复率、满意率分别为100%、99.8%。政府与法院、检察院、工会联席会议制度更加完善。机关效能建设、行政监察和审计监督不断加强。各位代表，2016年各项任务的完成，标志着本届政府任期目标基本实现。过去的五年，是龙岩发展历程中极不平凡的五年，习近平总书记两次对长汀水土流失治理作出重要批示，亲自决策并主持的“新古田会议”在我市召开，中央、省出台促进苏区老区振兴发展的政策，给我们极大的鼓舞和支持；我们主动适应经济发展新常态，积极应对增速换档、结构调整、动能转换带来的困难和挑战，危中求机、稳中求进，各项事业取得长足进步，谱写了龙岩发展新篇章。

五年来，经济发展迈上新台阶。人均地区生产总值突破1万美元，固定资产投资、社会消费品零售总额等主要指标实现年均两位数增长。有色金属、机械等传统产业优化升级，光电新材料等新兴产业及现代服务业快速提升，新模式、新业态孕育成长。企业创新能力和竞争力明显增强，新增上市企业10家、累计12家。三次产业结构由2011年的12.3：56.5：31.2调整为12.1：50.8：37.1。

五年来，人民生活实现新提升。城乡居民人均可支配收入年均分别增长9.9%和10.5%，贫困人口减少14.8万人。完成造福工程危房改造3.7万户，建成保障性安居工程3.7万套，解决170.6万农村人口饮水安全问题。合作医疗实现市级统筹，在全省率先实现城乡居民养老保险和市（县）福利中心、乡镇敬老院全覆盖。全面完成义务教育发展基本均衡县创建，文化、卫生、体育等事业协调发展。

五年来，城乡建设展现新面貌。新增高速公路通车里程339公里、累计662公里，新增铁路171公里、累计625公里，高速公路和铁路密度分别为每百平方公里3.5公里和3.3公里，居全省前列。永定撤县设区，形成“一市多区”发展格局，中心城市建成区面积扩大13平方公里、达55平方公里。打造一批美丽乡村典型，共有106个村镇获得国家级、省级历史文化名镇名村等称号，位居全省前列。全市常住人口城镇化率提高5.6个百分点、达53.1%。

五年来，生态环境彰显新优势。成功创建国家园林城市、国家森林城市、全国绿化模范城市，顺利通过省级生态市考核验收。节约集约利用资源，连续17年实现耕地占补平衡，森林覆盖率提高到77.9%，保持全省首位。治理水土流失面积268万亩，水土流失下降率居全省首位，“长汀经验”在全国推广。重点流域水环境持续改善，城市环境空气质量居全省前列。

五年来，改革开放取得新成果。行政审批制度、基层医药卫生体制、集体林权制度、“三农”综合保险、不动产统一登记、关检合作“三个一”等改革走在全国、全省前列。与厦门共建全省首个山海协作经济区，龙岩陆地港建成投入使用。一批优势企业加快“走出去”，国际友好城市合作不断拓展。

此外，国防动员和后备力量建设成效明显，“双拥”共建持续深化。人事、统计、民族宗教、外事侨务、库区移民、水文、气象、地震、地灾防治、人防、档案、地方志、科普、邮政管理、无线电管理、对口援建、老年人、妇女儿童、青少年、残疾人以及慈善事业等工作进一步加强。

各位代表，成绩来之不易，这是省委、省政府和市委正确领导的结果，是全市上下团结一心、奋力拼搏的结果，是各位市人大代表、政协委员和社会各界大力支持的结果。我代表龙岩市人民政府，向全市人民，向各民主党派、工商联、各人民团体和各界人士，向中央和省驻岩单位、驻岩部队和武警官兵，向关心支持龙岩发展的港澳台同胞、海外侨胞和国际友人，表示衷心的感谢！

同时，我们清醒看到前进中的困难和挑战：我市经济总量仍然较小，产业结构不合理，自主创新能力不强，产品供给质量不高；新旧动能转换接续不快，一些行业、企业生产经营困难，面临稳增长、促转型的双重压力；发展不平衡、不协调问题仍然突出，城市规划建设管理有待加强，农村基础条件比较薄弱，脱贫攻坚任务艰巨，生态环保压力较大；基本公共服务和社会保障水平不高，社会治理存在薄弱环节，一些群众关注的热点、痛点问题尚未得到有效解决；政府职能转变还不到位，一些干部服务意识、创新意识、担当意识不强，存在不作为、不善为、慢作为的现象。对此，我们将坚持问题导向，下决心解决。二、新一届政府工作目标任务和2017年工作建议

今后五年，是全面建成小康社会的决战决胜期。我们将认真贯彻落实市第五次党代会提出的“再上新台阶、建设新龙岩”各项决策部署，扎实推进各项工作。

今后五年，政府工作的总体要求是：高举中国特色社会主义伟大旗帜，全面贯彻落实党的十八大以来党中央一系列决策部署，深入贯彻习近平总书记系列重要讲话精神和来闽来岩重要讲话精神，紧紧围绕“五位一体”总体布局和“四个全面”战略布局，牢固树立和践行新发展理念，认真落实市第五次党代会部署，以推进供给侧结构性改革为主线，全面实施“三大战略”，深入打好“三大战役”，全力建设创新型工贸旅游强市，为加快建设机制活、产业优、百姓富、生态美的新龙岩，全面建成小康社会而努力奋斗。

今后五年，要全面完成“十三五”规划确定的各项任务，顺利实现市第五次党代会确定的“一个确保、五个突破、五个再上新台阶”目标，在建党100周年前全面建成小康社会，经济总量、综合实力显著提升，改革创新、城乡发展、生态环保、人民生活水平等方面取得新进展，实现更高质量、更有效率、更加公平、更可持续的发展。

围绕实现上述目标，我们将突出创新驱动，以科技创新为核心带动全面创新，加快集聚各类高层次人才，推动大众创业万众创新，以高效率的创新体系支撑高水平的创新型龙岩建设。突出项目带动，围绕发展抓项目，围绕项目抓落实，坚持工作项目化、项目责任化，滚动推进重大项目建设，扩大有效投资，强化发展支撑。突出结构优化，从供给侧发力，推进产业转型升级，培育形成3个千亿级产业集群、10个百亿级领军企业、10个百亿园区，基本建成现代工业体系和现代服务业体系。突出脱贫攻坚，现行标准以下贫困人口全部脱贫，城乡基本公共服务均等化水平进一步提高，建成公平可持续的社会保障体系，不断增进民生福祉。突出生态宜居，统筹生产、生活、生态布局，加快以人为核心的新型城镇化，全面建设国家生态文明试验区，构建功能互补、特色鲜明、环境优美、布局合理的现代化城镇体系。突出安全稳定，深化社会治理创新，推进平安龙岩、法治龙岩、诚信龙岩建设，社会更加安定和谐。

各位代表，2017年是新一届政府履职的第一年，是实施“十三五”规划的重要一年，挑战与机遇并存。我们将按照中央、省、市经济工作会议精神，坚持稳中求进工作总基调，全面做好稳增长、促改革、调结构、惠民生、防风险各项工作，促进经济社会平稳发展。

今年发展主要预期目标是：全市地区生产总值增长8.6%；一般公共预算总收入增长3%，地方一般公共预算收入增长6%；全社会固定资产投资增长16%；社会消费品零售总额增长12%；外贸出口占全省的份额稳中有升，实际利用外资增长6%；城镇居民人均可支配收入增长9%，农民人均可支配收入增长9%；居民消费价格总水平涨幅控制在3%左右；城镇登记失业率控制在4.2%以内；完成省下达的节能减排降碳任务。在实际工作中，力争完成得更好更快些。为此，重点做好以下六个方面工作：

（一）着力创新驱动，加快动能转换

1．深入推进“三去一降一补”。稳妥有序关闭年产9万吨以下地方煤矿，淘汰煤炭落后产能128万吨以上。鼓励合理自住购房和农民进城购房，培育发展住房租赁市场，推动商业地产根据市场需求转型，缩短非住宅类商品房库存去化周期。鼓励金融机构加大对实体经济支持力度，推动企业市场化、法治化债转股，力争新上市1家以上、“新三板”挂牌5家以上。切实减轻企业税费负担，降低企业物流、融资、用能、人工等成本，促进企业降本增效，激发实体经济市场活力。完善“一包一策”和“五个一”推进机制，策划实施更多投资工程包，加快补齐短板，增加公共服务和产品供给。

2．持续推进改革。深化“放管服”改革。推行“互联网+政务服务”，探索行政审批事项全市范围内跨区域办理，实现“一站式”受理、“全流程”服务，让群众和企业好办事、不添堵、少跑腿。充分激发市场活力。落实“先照后证”“五证合一”“一照一码”制度，全面推行“双随机一公开”，让企业充分享受市场经营的自主权和获得感。深化国资国企改革，完善治理、强化激励、突出主业，增强国有经济活力和竞争力。深化农村产权制度改革，完成农村土地承包经营权确权登记发证，推进农村土地“三权分置”，鼓励农业适度规模经营。抓好社会事务改革。加快公立医院综合改革，完善分级诊疗制度，鼓励和规范社会力量办医。完成生态文明试验区建设重点改革任务，开展林业碳汇交易、汀江-韩江跨省流域生态保护补偿、长汀自然资源资产负债表编制等试点工作，争取在资源资产产权制度、绿色发展绩效考核等方面先行示范；培育环境治理和生态保护市场主体，加快发展绿色环保产业，探索形成生态文明建设“龙岩模式”。

3．增强创新能力。强化企业创新主体地位，加大对科技型初创企业、科技小巨人领军企业扶持力度，支持小微企业创新活动，力争研发经费投入增长15%，新认定高新技术企业10家、科技小巨人领军企业10家以上。鼓励企业与科研院所联合开发产学研合作项目，组建科技创新重点战略联盟，加快战略性新兴产业创新平台建设，争取新建省级企业工程技术研究中心2家、重点实验室1家、众创空间5家以上。建立完善科技项目收益分配、科技成果处置收益、知识产权保护等制度，探索实行股权激励、分红激励等办法，加快科技成果转化应用。

4．激发人才活力。实施"121"高端人才引进培养工程和机关事业人才引进“千人计划”，创新柔性引才引智模式，加快引进一流人才。建立完善海外高层次人才引进工作站、北京高层次医疗卫生专家精准帮扶工作站等平台。推行产教融合、校企合作，加强龙岩学院、闽西职业技术学院等示范性院校和实训基地建设，培养优秀实用型人才。建立完善人才流动、人才评价激励、人才服务保障机制，鼓励科研人员创新创造，实施大学生返乡创业、农村青年创业富民行动，保护企业家精神，让一切人才的知识、技术和创业创新活力竞相迸发。

5．以开放促创新。对接国家“一带一路”和省21世纪海上丝绸之路核心区建设，加大“引进来”与“走出去”步伐，推动更多企业参与国际合作，增创竞争新优势。加强与厦门市和福建自贸区的对接，完善厦龙经济区共建机制，创新管理方式，实现山海互动、优势互补。提高龙台合作水平，深化产业对接和特色文化、民间基层交流。加强龙港龙澳经贸合作及赣州、梅州等周边区域协作，推进闽赣产业合作区建设、跨区域生态保护。加强国际友城交流，拓展开放新空间。扎实做好对口援建工作。

（二）着力项目带动，扩大有效投资

1．持续推进“五个一批”。围绕"365"产业发展和补齐短板，推动项目滚动接续，全年新谋划项目200个，新签约项目200个，新开工项目80个以上，新投产项目80个以上，新增资项目50个以上，全社会固定资产投资完成2600亿元以上。

2．全力抓好重大项目。全年安排300个省市重点项目，完成投资705亿元以上。全力攻坚30个“重中之重”产业项目，突出抓好龙岩锂离子动力电池、新龙马年产10万辆乘用车技术改造等重大项目。全力攻坚15个“重中之重”基础项目，突出抓好南三龙铁路、福广高铁龙岩段、厦蓉高速扩建工程龙岩段、漳武线南靖至永定高速永定段、龙岩一中分校等重大项目。全力攻坚15个“重中之重”城建项目。

3．健全项目落地攻坚机制。每个项目一名领导协调指挥、一个工作专班负责运作、一套政策激励、一笔经费保障，建立健全正向激励、定期协调、督查通报等推进机制，强化资金、用地、审批等要素保障，强化一线考评干部，形成全社会齐心协力抓项目、支持项目建设的浓厚氛围。完善招商引资优惠政策和奖惩办法，开展定位、定点、定向精准招商，精心办好“11·8”机械博览会等经贸活动，争取在引进先进制造业和现代服务业重大项目上有新突破。优化PPP模式，运作产业股权投资基金，鼓励更多社会资本投入文化旅游、基础设施、公用事业、扶贫开发等领域。（三）着力结构优化，推进产业升级

1．培育壮大工业产业。实施九大工业重点产业行动计划，推动产业加快向中高端迈进。做大做强主导产业。发挥紫金矿业、紫金铜业龙头带动作用，拉长铜、稀贵金属、循环经济产业链，发展稀土新材料及应用产业，力争有色金属产业实现产值465亿元以上。以龙工、龙净环保、龙马环卫、新龙马等企业为龙头，加快打造工程机械、环保装备、新能源汽车和专用车全产业链，推广“制造业+服务”，力争机械制造产业实现产值350亿元以上。培育发展新兴产业。推进智能终端产品、光学板材加工、医疗器械产业园等项目建设，推动光电与新材料、生物医药等新兴产业延长产业链、提升价值链。规划建设云计算大数据产业园，带动发展下游应用产业。推进军民深度融合发展，创建军民融合国家新型工业化产业示范基地和全国应急产业示范基地。改造提升传统产业。以100项重点工业技改项目为抓手，推动纺织、建材等传统产业加快技术装备更新、智能化改造和新产品开发。实施百亿企业培育工程、中小微工业企业培育计划，全年新增规模以上工业企业100家、亿元企业30家以上，培育一批百亿企业。

2．加快发展现代服务业。全面实施现代服务业11个重点行业三年行动计划，推进“五大幸福产业”服务消费提质扩容，力争2017年服务业增加值占GDP比重达到39%。文化旅游产业。积极引进知名企业，设立产业基金，加强资源整合、景区提升、产品开发、品牌培育和业态融合，建设以大古田旅游区为龙头的红色文化旅游长廊和以大客家文化为纽带的闽粤赣客家旅游文化长廊，推进珠宝文化产业园、文化创意产业园等项目，发展文化消费、动漫游戏等新业态，打造文化旅游全产业链，争取文化旅游产业收入500亿元以上。商贸物流业。重点推进龙岩公路港等项目建设，争取设立陆地港保税物流中心和跨境快件监管中心，大力发展冷链物流、第三方物流、快递服务业等现代物流业。加强线上线下互动，发展体验消费、新零售，推动实体店转型，推进商贸流通业创新发展。健康产业。加快构建以居家为基础、社区为依托、机构为补充、医养结合的多层次、全覆盖的养老服务体系，大力发展养老产业。发挥体育资源和品牌优势，培育发展体育产业。教育培训产业。充分发挥古田会议品牌效应，以古田干部学院为依托，整合各类红色资源和教学资源，打造全国党员干部教育基地。规划建设现代物流、旅游休闲、电子商务等现代服务业集聚区，争取创建1家以上省级服务业集聚示范区。

3．促进农业提质增效。深入推进农业供给侧结构性改革，推行绿色生产方式，狠抓农产品标准化生产、品牌创建、质量安全监管，增加绿色优质农产品供给。稳定粮食生产，落实粮食播种面积273万亩，总产量稳定在100万吨以上。以闽西“八大干”“八大珍”“八大鲜”为重点，加强地方特色农产品保护开发，加快发展设施农业、精致农业、生态养殖业，做大林下经济、花卉苗木等特色产业。深入实施农产品加工产业行动计划，推进种养加一体、农业“接二连三”融合发展，提高农业全产业链效益和竞争力。

（四）着力脱贫攻坚，加强和改善民生

1．推进精准脱贫。落实脱贫责任。健全挂钩帮扶、资金运作、督查考评和贫困人口“进、退”动态管理等制度，统筹扶贫资源，做好脱贫后续保障，提高脱贫的质量和可持续性，确保年度减贫5万人以上、现行国定标准以下贫困人口全部脱贫、造福工程扶贫搬迁3万人以上。落实脱贫项目。有效运作扶贫小额信贷平台和扶贫开发融资平台，帮扶每个有劳力的贫困户发展好1个以上的种植、养殖、加工、电商、服务业等脱贫增收项目，加快构建县级有龙头企业带动、乡镇有特色产业支撑、村级有合作组织运作、贫困户有生产项目增收的产业扶贫体系。加快易地扶贫搬迁，建设10个百户以上、10个50户以上省级集中安置区和30个市级集中安置区。大力推进长（汀）连（城）武（平）扶贫开发试验区建设，加大对革命基点村、民族村等扶持力度。落实脱贫政策。推进教育扶贫，确保每个贫困家庭的孩子不因学费问题而失学。实施健康扶贫行动计划，提高贫困人口医疗费用报销比例，加大医疗救助力度，探索试行商业医疗保险，减少贫困人口因病致贫、返贫。整合各类扶贫资金，探索农村资产收益扶贫、涉农项目投资收益扶贫等新模式。鼓励和支持企业、社会组织、个人参与扶贫开发，合力打赢脱贫攻坚战。

2．扎牢民生保障网。要把群众的安危冷暖时刻挂在心上，对特殊困难群体要格外关心、格外关注，多为他们分忧解难。实施就业促进计划和创业引领计划，加强企业用工服务，重点抓好高校毕业生、农民工、就业困难人员等群体就业，全年新增城镇就业1.9万人。扩大社会保障覆盖面，将符合条件的各类群体纳入社会保障制度覆盖范围，基本医保制度实现全市统筹，推进机关事业单位养老保险制度改革。提高城乡低收入家庭保障水平，实行农村低保与国定贫困标准“双线合一”，加大对特殊困难群体的救助。大力发展志愿服务、社会福利和慈善事业。建立完善社会救助体系和农村留守儿童、妇女、老年人关爱服务体系。

3．改善基本公共服务。解决群众热点痛点问题。全力推进畅通工程，优先发展公共交通，开展城市交通堵点综合整治，新增公共停车位1500个以上，投放一批城市公共自行车，改造提升农村公路230公里，努力解决“出行难、停车难”问题。深化“餐桌污染”治理，建立覆盖从田间到餐桌全过程、可追溯的监管体系，全面推进食品药品安全放心市建设，确保“舌尖上的安全”。建好保障性安居工程，新开工建设4000套、建成3500套。提高社会事业发展水平。全面实施中心城区教育重点项目建设三年行动计划，开工建设28个项目；全面改善农村义务教育薄弱学校基本办学条件，新建11所公办幼儿园，扶持发展普惠性民办幼儿园；实施教育质量提升行动计划，推进教育强县、教育名城创建；规范和支持民办教育发展。推进健康龙岩建设，加快补齐产科、儿科、康复、老年护理等短板，加强乡村医生队伍建设，缩小城乡基本卫生与健康服务差距。广泛开展全民健身活动，办好龙岩市第十五届运动会。推进国家级客家文化（闽西）生态保护实验区建设。加强闽西中央苏区革命旧址群保护与开发。完成70%的基层综合文化服务中心建设，深入开展“文化下乡”等活动。做好“双拥”工作。落实好拥军优属政策，抓好国防动员和后备力量建设，支持武警、消防部队建设，努力实现创建全省双拥模范城（县）“满堂红”。（五）着力生态宜居，推进城乡协调发展

1．强化环境治理。深入实施水、大气、土壤污染防治行动计划，全面落实省环保督察整改要求，下大力气解决环境突出问题。强力推进水环境整治。全面推行“河长制”，落实最严格水资源管理制度，深入开展九龙江、汀江、闽江等重点流域及50条小流域综合治理，坚决清理整治非法排污、设障、养殖、采砂等活动，抓好畜禽养殖、污水垃圾等污染专项整治，推进畜禽养殖废弃物处理和资源化。实施万里安全生态水系、防洪工程、蓄水工程、引调水工程、烟草援建水源工程、城市内涝防治等一批水利工程。狠抓大气污染防治。推进重点企业清洁生产和工业园区循环化改造，开展道路扬尘、矿山扬尘、垃圾焚烧等城市面源污染治理，淘汰黄标车5269辆，全部淘汰10蒸吨及以下燃煤锅炉，确保空气质量保持全省前列。持续提升“长汀经验”。加强生态修复，稳步推进二重山、三重山和山顶、山脊等区域水土流失治理，确保完成治理面积40万亩，坡耕地综合整治13万亩。加强危险废物和土壤污染治理，抓好马坑太保林矿区、东宫下高岭土矿区和龙津河流域等矿区生态修复治理。推进湿地公园建设，加强生态公益林、天然林保护，完成造林绿化11.5万亩、封山育林43万亩。

2．提升城市规划建设管理水平。高标准编制完善各类规划，加快推进“多规合一”，统筹开发地上地下空间，强化土地节约集约利用。做活“山”“水”文章，提升交通、市政、旅游、工业园区配套、社区服务等专项规划，打造文脉传承、天人合一的宜居宜业宜游城市。推动永定区、经开区加快融入中心城市，在产业协同发展、基础设施对接、公共服务共享、体制机制创新等方面迈出更大步伐，加快市区一体化联动发展。集中力量攻坚龙津湖片区改造、龙岩大道高架桥、龙岩大道北段四期、北三环、罗龙路改移等一批“重中之重”城建项目，积极推进东肖新城开发和城中村改造。建立城市管理服务的长效机制，强化职能整合、部门联动，对违法建设、交通秩序、建筑渣土、占道经营、环境卫生等进行常态化整治，提高城市精细化、智能化管理水平，让城市更美观、更宜居。

3．打造特色小镇和美丽乡村。推进古田红色小镇、永福花香小镇、培田草药小镇等特色小镇建设，支持各县（市、区）、经开区分别打造1个以上休闲旅游、民俗文化传承、先进制造、创客集聚等特色小镇。实施第二批124个市级美丽乡村建设和第四批省级“千村整治、百村示范”工程，整治村庄100个以上，培育美丽乡村示范村12个。深化农村家园清洁行动，全面完成380个农村垃圾治理和550个农村改厕任务，健全农村卫生保洁长效机制，开展乡风文明建设，打造文明村镇。

（六）着力安全稳定，构建平安和谐的社会环境

1．创新社会治理。建设更高水平的平安龙岩。推进法治龙岩建设，开展“七五”普法，建立健全公共法律服务体系。认真履行综治维稳责任，落实综治激励惩戒机制，推进互联网+社会综合治理，建设网格化服务管理信息平台。健全社会治安立体化防控体系，实施“雪亮工程”；严厉打击各类违法犯罪活动，全力抓好涉麻制毒、电信诈骗、涉爆涉矿等突出问题整治。健全社会矛盾纠纷多元化解机制，建立专业调解组织，及时有效排查和化解矛盾纠纷。依法处置信访事项，落实“三到位一处理”。推动社会组织多元健康发展，发挥工会、共青团、妇联等群团组织在社会管理服务中的积极作用。加强城乡社区服务机构和人才队伍建设，提升社区公共服务水平。

2．防范化解风险。强化红线意识，完善“党政同责、一岗双责、失职追责”安全生产责任体系，夯实安全生产基层基础，加强对道路交通、民爆器材、矿山、人员密集场所等重点行业、重点领域的监管和整治，坚决遏制重特大事故发生。加强对饮用水源、矿区、污水垃圾处理等环境监管，抓好尾矿库“头顶库”整治，严防发生环境污染事故。完善灾害监测预警应急体系，增强防灾减灾救灾能力。加强政府债务风险管控，加快建立“借、用、还”相统一的地方政府举债融资新机制。严厉打击非法集资等非法金融活动，促进民间金融和互联网金融规范发展，有效防范和及时处置企业信贷风险。

3．创建文明城市。紧紧围绕争创全国文明城市，深化群众性精神文明创建活动，培育社会主义核心价值观，传承和发扬红色文化、客家文化、闽南文化，加强未成年人思想道德建设，引导市民自觉遵守社会公德、职业道德、家庭美德和个人品德，提高文明素质，让文明成为一种习惯。加强社会信用建设，强化守信激励和失信惩戒，形成诚信守信的社会风尚。实施社区基础设施提升工程包，加强城区农贸市场、公厕、人行通道、绿道等市政公用设施建设，打造城市文化景观，进一步美化市容市貌。三、建设人民满意的政府

牢固树立政治意识、大局意识、核心意识、看齐意识，坚决维护以习近平同志为核心的党中央权威，不折不扣把中央、省委省政府和市委的各项决策部署落到实处，对人民负责、受人民监督、让人民满意。

勤政务实。坚持发展第一要务，围绕发展抓产业，围绕产业抓项目，围绕项目抓服务。坚持实事求是，坚持“三严三实”，以实绩论英雄，以实干促发展，以“钉钉子”的精神一抓到底、抓出成效。继承和发扬苏区干部好作风，一心一意为人民服务，以人民群众满意不满意衡量工作实效。加强机关效能建设，大力倡导“马上就办、真抓实干”，坚决克服“不冷不热、不急不忙、不疼不痒”现象，真正服务好企业、服务好基层、服务好群众。

廉洁干净。加强党风廉洁建设，严格遵守《中国共产党廉洁自律准则》《中国共产党纪律处分条例》和中央八项规定精神，严格落实“五抓五看”要求，不触底线、不逾红线。坚决反对以权谋私，深化政务公开，加大行政监察和审计监督力度，加强土地出让、工程建设、产权交易、政府采购等重点领域廉洁风险防范，构建“亲”“清”新型政商关系。

勇于担当。以改革的思维和创新的办法破解发展难题，遇到问题不回避、面对阻力不退缩，多为闽西发展尽心尽责、冲锋在前、攻坚克难。建立正向激励机制，为敢于担当的干部担当、为敢于负责的干部负责，营造干事创业、比学赶超的浓厚氛围。

依法行政。把政府工作全面纳入法治轨道，办事依法、遇事找法，解决问题用法、化解矛盾靠法，自觉运用法治思维和法治方式推动工作，真正做到法定职责必须为、法无授权不可为。自觉接受市人大及其常委会的法律监督、工作监督和市政协的民主监督，欢迎社会公众监督和舆论监督。

各位代表！新起点、新征程、新使命。让我们紧密团结在以习近平同志为核心的党中央周围，在中共龙岩市委的坚强领导下，不忘初心、继续前进，团结一心、真抓实干，全力建设创新型工贸旅游强市，再上新台阶、建设新龙岩，以优异成绩迎接党的十九大胜利召开！
